# Imine Resveratrol Analogues: Molecular Design, Nrf2 Activation and SAR Analysis

**DOI:** 10.1371/journal.pone.0101455

**Published:** 2014-07-16

**Authors:** Chang Li, Xiaofei Xu, Xiu Jun Wang, Yuanjiang Pan

**Affiliations:** 1 Department of Chemistry, Zhejiang University, Hangzhou, P. R. China; 2 Department of Pharmacology, School of Medicine, Zhejiang University, Hangzhou, P. R. China; North Carolina State University, United States of America

## Abstract

Resveratrol is a natural phenol with protective effects against cancer and inflammation-related diseases. Its mechanism of action involves the activation of nuclear factor E2 p45-related factor 2 (Nrf2), which plays a key role in regulation of genes driven by antioxidant response element (ARE). Inspired by the effect of resveratrol, here we synthesized a series of imine resveratrol analogs (IRAs), evaluated their abilities to activate Nrf2 by using cell based ARE-reporter assay. After the first-round screening, preliminary and quantitative structure-activity relationship (SAR) was analyzed, and the structural features determining Nrf2 activation ability were proposed. Two novel IRAs were designed and subsequently synthesized, namely 2-methoxyl-3,6-dihydroxyl-IRA and 2,3,6-trihydroxyl-IRA. They were proved to be the most potent Nrf2 activators among the IRAs.

## Introduction

Chemoprevention of cancer, which is a strategy using natural or synthetic small molecules to modulate the metabolism, can reverse, suppress, prevent or delay the process of carcinogenesis [1], [2]. The preventive effect could be achieved either by disposition of endogenous and exogenous carcinogens directly, or through upregulation of phase II/III enzymes which can deactivate toxic reactive chemical species. The nuclear factor (erythroid-derived 2)-like 2 (Nrf2), which has emerged as a key regulator of the cancer-preventive genetic program, can regulate defensive enzymes through antioxidant response elements (AREs) [3], [4]. These enzymes include phase II/III enzymes (e.g. NQO1, NAD(P)H: quinone oxidoreductase 1) and stress-response proteins (e.g. HO-1, heme oxygenase 1).

Under basal condition, Nrf2 is sequestered in cytoplasm by its major repressor, Kelch-like ECH-associated protein 1 (Keap1) which mediates ubiquitin-mediated degradation of Nrf2. In contrast, electrophile/ROS (reactive oxygen species) can covalently modify Keap1 and further induce release of Nrf2 in stressed cells. Such modifications, which include oxidation of key cysteine residues in Keap1, can lead to the disruption of Keap1–Nrf2 complex. Nrf2 hereby migrates to the nucleus, binds to AREs in the promoters of its target genes, and increases their transcription [5]–[7]. This process can further contribute to the protection of cells against oxidative stress.

Developing small molecules with Keap1 modifying properties has been considered as a valid strategy to achieve chemoprevention of cancer [8]. Both natural (e.g. sulforaphane [9], EGCG [10] and curcumin [11]) and synthetic small molecules (e. g. butylated hydroxyanisole (BHA) [12], *tert*-butylhydroquinone (tBHQ) [13] and olitipraz [14]) have been developed as Nrf2 activators in recent years. Among the synthetic approaches, Kumar *et. al*. [15] reported a series of trifluoromethyl-bearing chalcone derivatives based on cell and mouse model tests. Lee *et. al*. [16] developed hydroxyl substituted sulfuretin analogs as potent Nrf2 inducers. Very recently, Vrba *et. al*. [17] discovered that a synthetic flavonoid, 7-O-galloyltaxifolin greatly enhanced the expression of HO-1. Other potent Nrf2-ARE inducers developed included 3-phenyl-1-alpha-pyrones [18], sulforaphane analogs] [19] and a cyano enone (TBE-31) [20].

On the other hand, resveratrol (3,4,5-trihydroxy-trans-stilbene) is abundantly distributed in several dietary sources, such as grapes (as well as wine), berries and peanuts. It has been shown to elicit a broad range of effects that interfere signaling pathways involved in cell proliferation and/or cell death *in vitro* and *in vivo* [21]. Resveratrol has also been shown to be able to upregulate Phase II enzymes [22]–[24]. The induction likely involves the Keap1-Nrf2-ARE pathway. The activation of Nrf2 by resveratrol is thought to confer protection against phase I enzyme-activated carcinogens and associated carcinogenicity via the transactivation of antioxidant and phase II detoxifying enzymes. However, *in vivo* data indicated that resveratrol has extremely low bioavailability and rapid clearance from the circulation [25]. Therefore, in order to explore the potential of resveratrol as chemopreventive agent, it is a beneficial alternative approach to develop new resveratrol analogs that mimic its effects but with improved bioavailability and higher potency in activating Nrf2.

In prior studies, we synthesized an array of synthetic polyphenols, imine resveratrol analogs (IRAs), by replacing the C = C bond of resveratrol with C = N bond [26].The simple replacement make it much more easier to generate resveratrol analogs with different substitutions, which can provide broader possibility to develop new analogs possessing higher bioavailability and Nrf2 inducing activity without losing the fundamental properties of resveratrol. Evaluation of the antioxidant activity with chemical model systems revealed that these IRAs are effective DPPH (2,2-diphenyl-1-picrylhydrazyl) radical scavengers and selective singlet oxygen quenchers, but ineffective to react with hydroxyl radicals and superoxide anions. This indicates that, like resveratrol [27] and its oligomers (Pan et. al., unpublished data), IRAs retained the features as direct antioxidants. In this study, we employed a cell-based ARE-reporter assay to examine the ability of the IRAs in activating Nrf2 pathway. Below, we present the results of *in vitro* screening, SAR analysis and molecular design of IRAs as Nrf2 inducers.

## Experimental Procedures

### Materials and instruments

All chemicals were purchased at the highest commercial quality and used without further purification. Reactions were magnetically stirred and monitored by TLC. NMR spectra were recorded on a Bruker 500 MHz instrument. Mass spectroscopic data were obtained using Waters GCT Premier oa-TOF mass spectrometer. MCF7 cells were obtained from ATCC (Shanghai, China) and cultured as described previously [28]. All media supplements for cell culture were purchased from Invitrogen (Shanghai, China).

### Synthesis of IRAs

**Figure 1:**
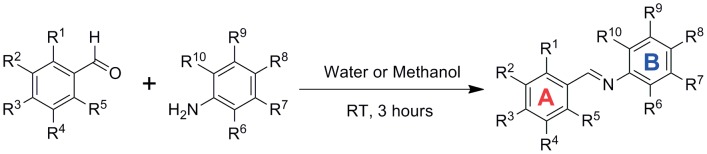
Synthesis of Imine Resveratrol Analogs (IRAs).

The process was carried out as described previously [26]. Briefly, differently substituted anilines and benzaldehydes in equal moles were added into small amount of water, forming a suspension. After stirred for 3 hours, the mixture was extracted with ethyl acetate twice. The organic layer was collected and evaporated in vacuum. The residue was then recrystallized in ethyl acetate or methanol twice. (**Figure 1**)

### ARE-reporter assay

A stable ARE-reporter cell line was developed after ARE-luciferase reporter plasmid pGL3-10xARE, which contains ten copies of the ARE (5′-GTGACAAAGCA-3′), was stably transfected into MCF7 cells. The cells were seeded in 96-well plates at a density of 1.2×10^4^ cells per well. After 24 h, culture medium was replaced with fresh DMEM supplemented with penicillin-streptomycin containing controls or IRAs (dissolved in DMSO to give a final 0.1% (v/v) of vehicle). After 24 h incubation, luciferase activities were measured. Three separate experiments were carried out in each case. The value for cells treated with vehicle DMSO (0.1% v/v) was set at 1. The values of tested compounds were presented as folds to vehicle DMSO control. Statistical analysis was done by unpaired Student's t tests, P<0.05 was considered statistically significant.

### Theoretical Calculation of QSAR Descriptors

24 different descriptors were calculated for each individual compound employing ChemBioDraw Ultra 12.0 or Gaussian 03W software package [29]. The calculated descriptors and corresponding software package were listed in **Table S1 in File S1** and the detailed results of theoretical calculation are listed in **Table S2 in** **File S1**.

### QSAR Statistical Analysis with SPSS

The regression analysis of obtained activities and calculated descriptors was carried out using IBM SPSS Statistics 19 software package. The Pearson correlation test was firstly carried out between activity and each individual descriptor, and those with correlation coefficients higher than 0.300 were selected as meaningful descriptors. Linear fitting was then carried out.

## Results and Discussion

### Initial Screening of IRAs and Preliminary SAR Analysis

The IRAs were prepared as described in previous work [26], with some modifications to Tanaka's method [30] (**Figure 1**). After recrystallization in methanol or ethyl acetate, the isolated yields ranged from 80% to 95%. Synthetic details and characterization data (^1^H NMR, ^13^C NMR and HRMS) are available in **Section I of File S1**.

The ARE-dependent firefly luciferase reporter cell assay, which has been used to assess transcriptional activation of Nrf2 previously [28], was employed to evaluate the ability of IRAs to activate Nrf2. The response of ARE-luciferase activity to 34 IRAs (32 first initial entries and 2 designed ones based on preliminary SAR analysis) were expressed as folds to DMSO control and presented in **Table 1**. In order to mimic the structure of resveratrol at the highest level, only hydroxyl, methoxyl and methyl groups were intensely considered.

**Table 1:** Synthesized IRAs and their effects on ARE-luciferase activity.

IRAs	Substituents on Ring A				Substituents on Ring B				Relative Fold to Control		
	R^1^	R^2^	R^3^	R^4^	R^6^	R^7^	R^8^	R^9^	at 7.5 µM	at 15 µM	at 30 µM
1	H	H	H	H	H	H	H	H	0.99±0.17	1.06±0.06	1.08±0.04
2	H	H	OH	H	H	H	H	H	0.78±0.12	0.83±0.05	0.88±0.08
3	H	OMe	H	OMe	H	H	H	H	0.97±0.10	0.92±0.31	1.06±0.25
4	H	OH	H	H	H	H	H	H	1.18±0.27	1.37±0.24	3.67±0.59
5	H	H	H	H	OH	H	H	H	1.83±0.24	3.03±0.13	3.91±0.18
6	OH	H	H	H	OH	H	H	H	2.36±0.32	3.56±0.71	4.71±0.92
7	H	OH	H	H	OH	H	H	H	2.58±0.16	4.16±0.44	8.90±1.20
8	H	OMe	H	H	OH	H	H	H	3.28±0.07	6.17±0.88	10.32±0.40
9	H	H	OH	H	OH	H	H	H	5.69±2.22	12.18±0.68	N.D.^a^
10	H	H	OH	H	H	OH	H	H	0.64±0.03	0.70±0.02	0.69±0.24
11	OH	H	H	H	H	OH	H	H	0.77±0.05	0.78±0.12	0.94±0.12
12	H	H	H	H	H	H	OH	H	1.11±0.13	1.41±0.27	2.78±0.45
13	OH	H	H	H	H	H	OH	H	1.25±0.17	1.79±0.26	4.62±0.46
14	H	H	OH	H	H	H	OH	H	0.96±0.20	1.19±0.07	2.50±0.48
15	H	OMe	H	OMe	H	H	OH	H	1.49±0.16	1.70±0.15	4.58±0.45
16	H	OH	OH	H	H	H	OH	H	0.98±0.16	1.42±0.14	3.14±0.29
17	H	OH	H	OH	H	H	OH	H	1.13±0.29	1.14±0.44	1.28±0.23
18	H	OH	H	H	Me	H	H	H	0.71±0.15	0.97±0.09	1.2±0.13
19	H	OMe	H	H	Me	H	H	H	0.99±0.10	1.06±0.03	1.08±0.09
20	H	H	OH	H	Me	H	H	H	1.07±0.02	0.92±0.03	1.05±0.10
21	H	OH	H	H	H	Me	H	H	0.97±0.05	0.97±0.05	1.31±0.12
22	H	OMe	H	H	H	Me	H	H	1.07±0.09	1.01±0.05	1.09±0.11
23	H	H	OH	H	H	Me	H	H	0.91±0.15	1.01±0.07	1.06±0.05
24	H	H	OH	H	H	H	Me	H	1.34±0.18	1.10±0.09	1.31±0.22
25	H	OH	H	H	H	H	Me	H	1.02±0.15	1.02±0.08	1.25±0.14
26	H	OMe	H	H	H	H	Me	H	1.13±0.10	1.03±0.05	1.20±0.03
27	H	H	Cl	H	H	H	Me	H	0.98±0.08	0.86±0.06	0.87±0.10
28	H	H	OMe	H	H	H	OMe	H	1.12±0.17	1.01±0.08	1.02±0.03
29	H	OMe	OMe	OMe	H	H	OMe	H	1.17±0.09	1.08±0.05	1.20±0.04
30	H	OMe	OMe	OMe	H	OH	OMe	H	1.07±0.08	1.04±0.04	1.03±0.07
31	H	OH	OMe	H	H	OMe	OMe	OMe	1.16±0.13	1.05±0.05	1.15±0.08
32	H	OH	H	H	Br	H	H	H	0.57±0.12	0.57±0.09	0.65±0.15
33	H	OMe	OH	H	OH	H	H	H	4.84±0.50	9.97±3.75	18.61±1.11
34	H	OH	OH	H	OH	H	H	H	6.93±1.55	12.33±4.11	14.28±0.97
resveratrol	-	-	-	-	-	-	-	-	2.54±0.22	3.05±0.62	5.10±2.11

ARE reporter cells were exposed to 7.5 µM, 15 µM or 30 µM IRA for 24 h. The value for cells treated with vehicle DMSO (0.1% v/v) was set at 1. Results are from three separate experiments.

a Due to cytotoxicity of **9**.

The preliminary Structure Activity Relationship (SAR) analysis was focused on the substituent effect on Ring B at the first step. Comparing **1** (without substitution), **5** (R^6^ = OH) and **12** (R^8^ = OH), it could be inferred that hydroxyl substitution on Ring B could enhance the Nrf2 induction activity at the concentration of 15 µM IRA (this concentration was used as standard index hereinafter) from 1.06 (**1**) to 1.41 (**12**) and 3.03 (**5**). Another inspiring comparison of compounds were **4**, **7**, **18**, **21**, **25** and **32**, which share a *meta*-OH substitution on Ring A but distinguish with each other on Ring B. Methyl substitution on Ring B in either position has no significant or even negative effect on the ARE-luciferase activity (comparing **18**, **21** and **25** with **4**), and bromo-substitution on Ring B showed negative effect (**32**). Meanwhile, an *ortho-*OH group in Ring B led to 3-fold increase of luciferase activity markedly. Other groups of data sets, as **2**, **9**, **10**, **14**, **20**, **23** and **24**, as well as **6**, **11** and **13**, could also support the same conclusion, that an *ortho-*OH group on Ring B in crucial for effective Nrf2 induction activity.

**Figure 2:**
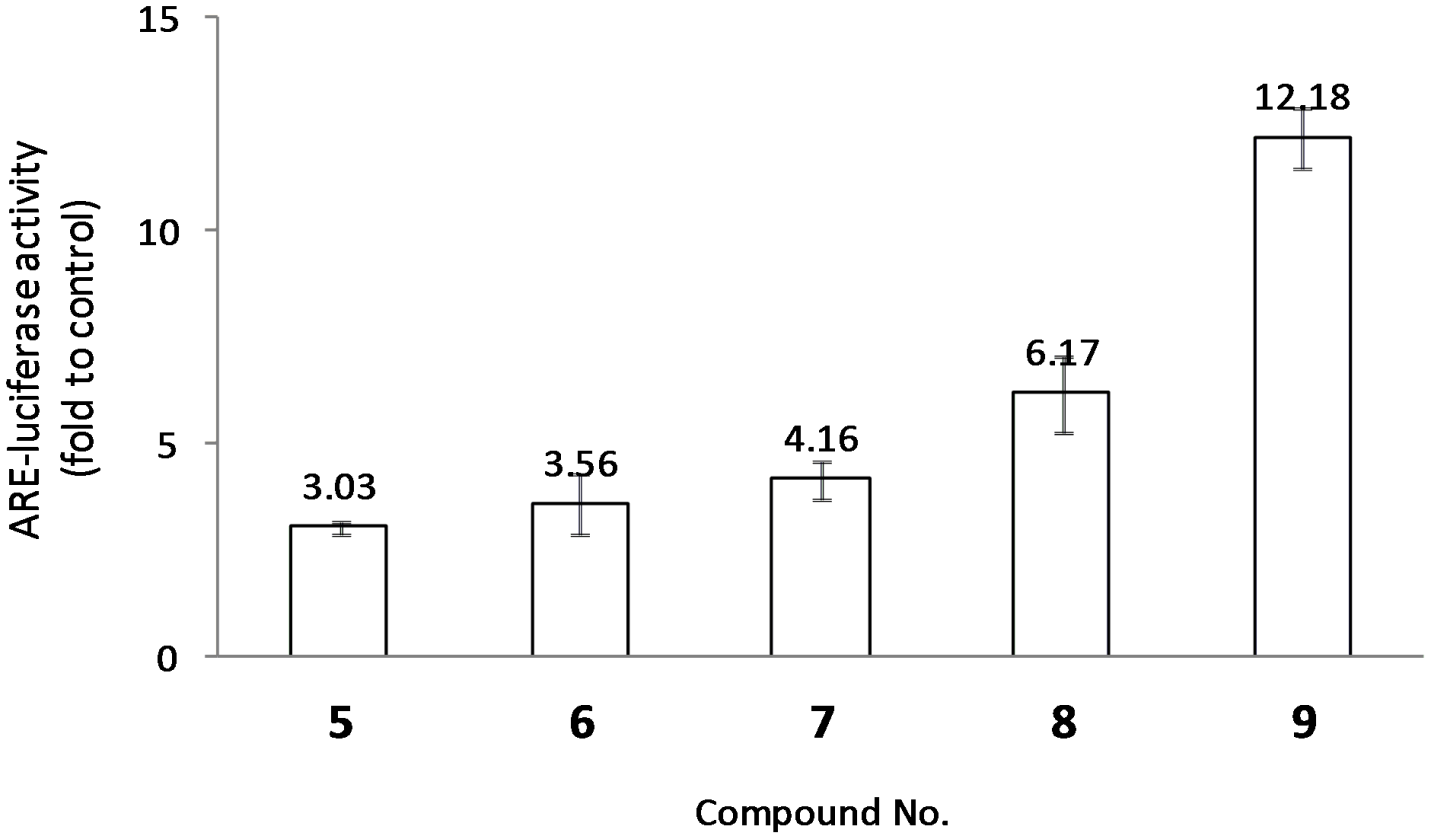
ARE-luciferase activities in response to 6-OH IRAs. ARE reporter cells were exposed to (15 µM) for 24 h. The value for cells treated with vehicle DMSO (0.1% v/v) was set at 1. Results are from three separate experiments.

**Figure 3:**
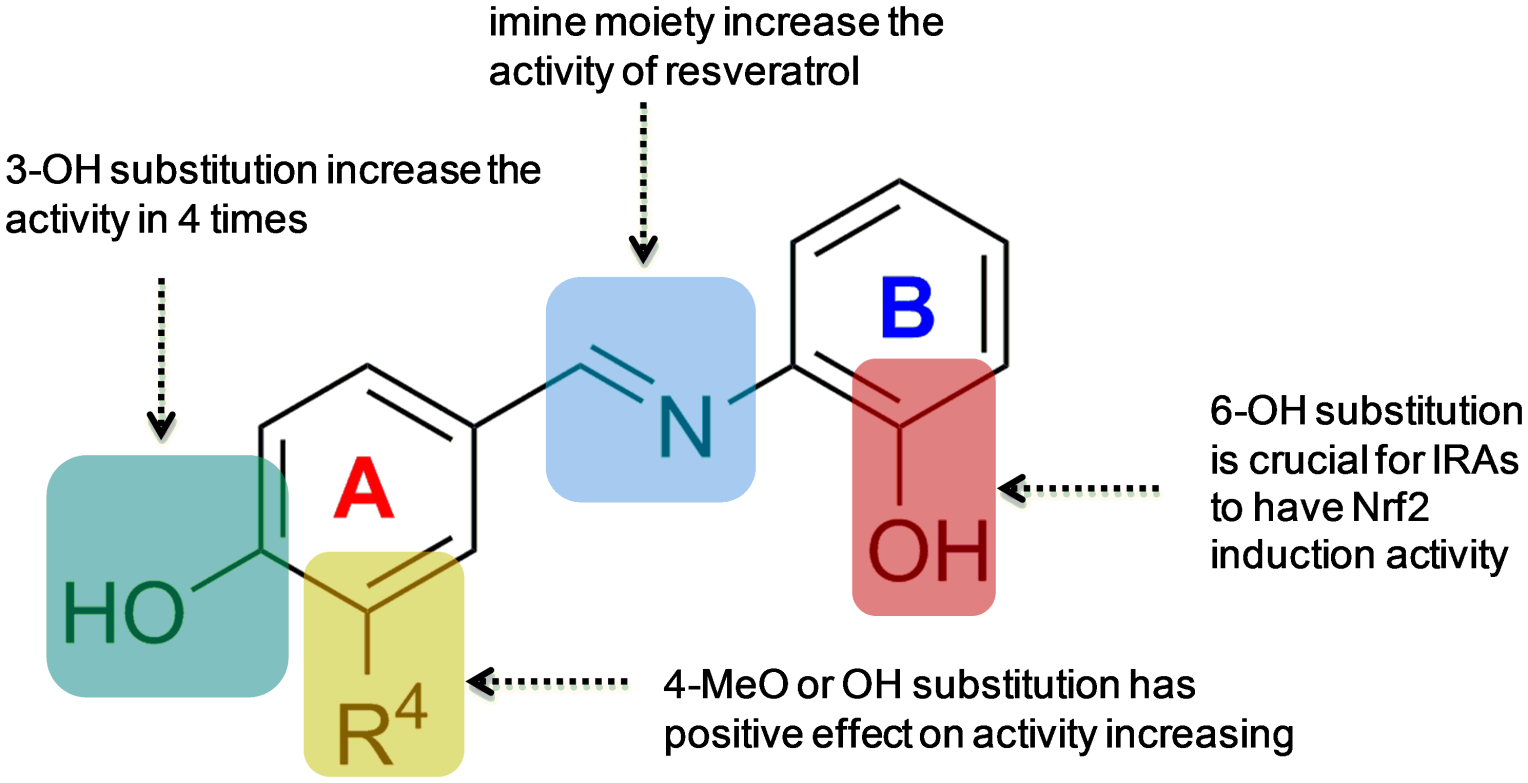
SAR results from initial screening of IRAs.

As *ortho-*hydroxyl group (R^6^ = OH) is a key substructure in optimization, further investigation was then concentrated on the substitution effect on Ring A. As shown in **Figure 2**, seven 6-OH IRAs with different substituents on Ring A were picked out. In comparison of IRA **5–9**, it could be apparently concluded that 3-OH (**9**) substitution is the most dominant activity increasing factor, followed by 2-OMe (**8**), 2-OH (**7**) and 1-OH (**6**) substitution. However, this conclusion could not be supported by data sets other than 6-OH substituted IRAs, **12–17** for instance. This phenomenon strengthened the conclusion that 6-OH substituent group plays the vital role in Nrf2 activation, while substitutions on Ring A contribute as auxiliary cofactors. The preliminary SAR results generated were summarized in **Figure 3**.

### QSAR Analysis

We then investigated the quantitative structure activity relationship (QSAR) of IRAs, as such an approach may reveal the mechanism of the interaction between IRA and Keap1, and possibly provide a statistic guide to design more effective IRAs. 24 different descriptors were calculated using either Gaussian 03W software package or ChemBioOffice Ultra 12.0 (presented in **Section II, Supporting Information**). After Pearson correlation test to all 24 descriptors (detailed results presented in **Table S3 in File S1**), it was found that the most correlated ones were Vertical Detachment Energy (**IP**), Energy of the Lowest Unoccupied Molecular Orbital (**E_LUMO_**), Energy of the Highest Occupied Molecular Orbital (**E_HOMO_**), Electrophilic Frontier Electronic Density (**Fr^E^**) and Calculated logP (**clogP**). At last, multiple linear regression (MLR) analysis was employed to search for suitable factors for aforementioned molecular descriptors. **Equation 1** was generated as follows (**LogA_15_** represents the Common logarithm of the activity of IRA at 15 µM concentration).
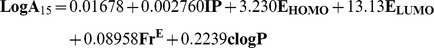
(**Equation 1**, correlation coefficient R  =  0.792, F-statics  =  9.417)

From the generated equation, it could be inferred that **E_HOMO_** and **E_LUMO_** are correlated with the Nrf2 induction activity to a great degree. As these descriptors are key variables in chemical reactions according to Fukui's Frontier Molecular Theory, this result can partly support the assumption that chemical reactions took place between IRAs and Keap1 protein. Moreover, **Fr^E^**, which also played an important role in this equation, is a descriptor describing the frontier electron density in electrophilic reactions. The aforementioned results both suggested a electrophilic substitution mechanism in this Nrf2 induction process.

### Molecular Design of Effective IRAs and Activity Evaluation

**Figure 4:**
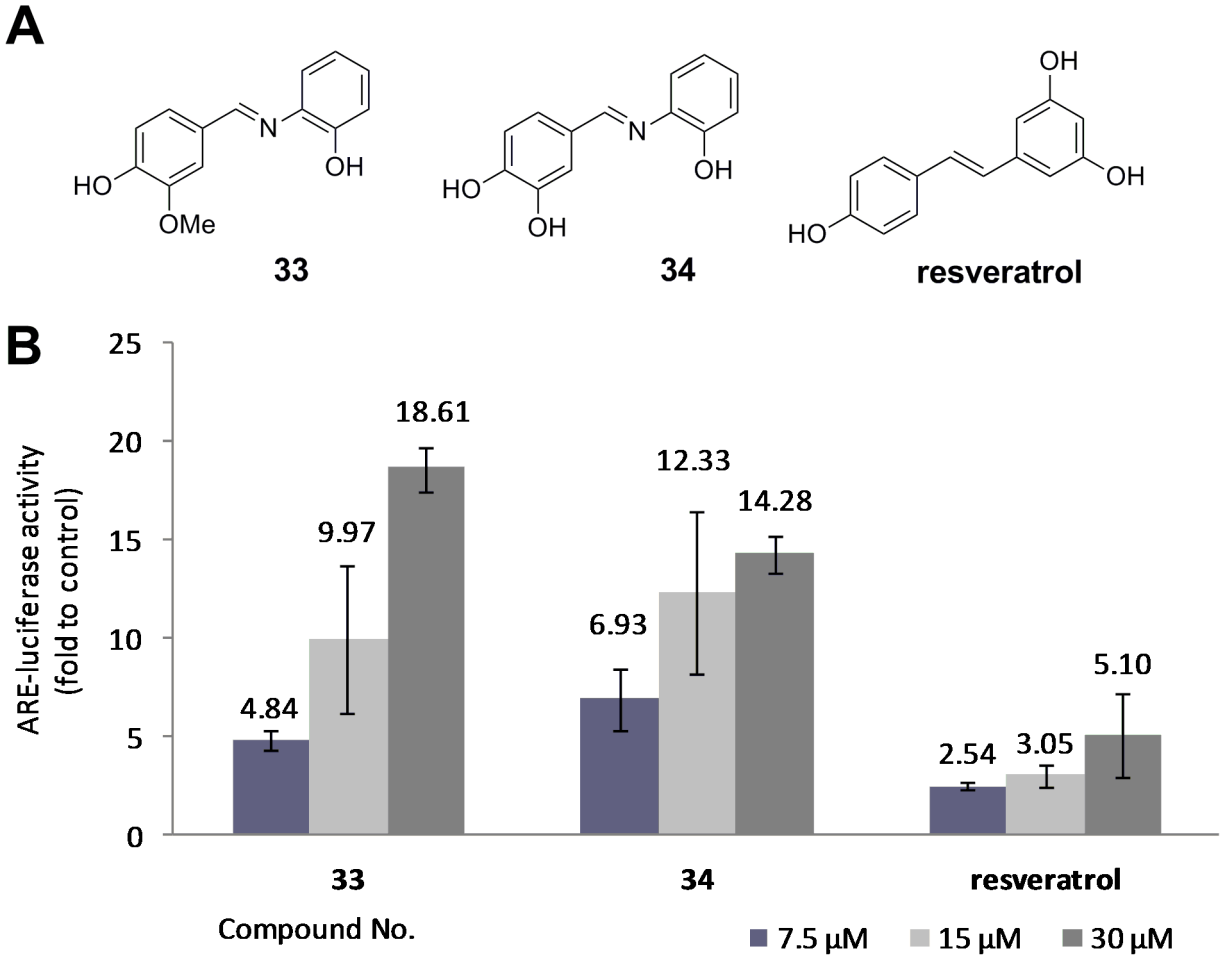
Structures (A) and ARE-luciferase activities (B) of 33, 34 and resveratrol. ARE reporter cells were exposed to **33**, **34** and resveratrol (7.5 µM, 15 µM and 30 µM) for 24 h. The value for cells treated with vehicle DMSO (0.1% v/v) was set at 1. Results are from three separate experiments. Values shown are mean ± SD.

Inspired by the preliminary and quantitative SAR results shown in **Figure 3**, two novel 6-OH IRAs, **33** and **34**, were designed and synthesized. As shown in **Figure 4A**, they are both bearing 3-OH substitutions, while the only difference was at 2-position, on which **33** has a methoxyl group and **34** has a hydroxyl group. ARE-luciferase assay revealed that **33** and **34** induced ARE-luciferase activity in a dose-dependent manner (the dose-dependency curve is shown in **Figure S1 in File S1**). Exposing the reporter cells to 7.5 µM compound **33** for 24 h, ARE-luciferase activity was elevated by nearly 5 folds, whereas that at 15 µM and 30 µM increased the luciferase activity 10 and 19 folds, respectively (**Figure 4B**). Similarly, 7.5 µM of compound **34** induced ARE-luciferase activity by nearly 7 folds, while 15 µM of the compound stimulated more than 12-fold induction of luciferase activity. This is much higher than that of their parent compound resveratrol, whereas under the same culture condition, 30 µM of resveratrol only achieved ∼5-fold induction of ARE-luciferase activity (also see **Table 1**). Besides, not like some other Schiff bases, **33** and **34** are stable in a blank ARE luciferase assay condition. However, their radical scavenging activities are poor, 500 µM of these compounds can only scavenge 4.0% and 9.7% DPPH radical, while resveratrol can achieve complete quench in these concentrations. Therefore, our data demonstrate that **33** and **34** are the most potent Nrf2 activators among the resveratrol analogs.

Most Nrf2 activators, despite the structural diversity, are all electrophiles that have striking propensities to react with sulfhydryls [31]. Here our theoretical calculation results of Electrophilic Frontier Electronic Density indicate that compound **33** and **34** are reactive electrophiles. As Nrf2 inducers implement their functions by forming covalent bonds with thiol groups of Keap1 [3c], we therefore also classify the IRAs as inhibitors of Keap1-Nrf2 interaction.

**Figure 5:**
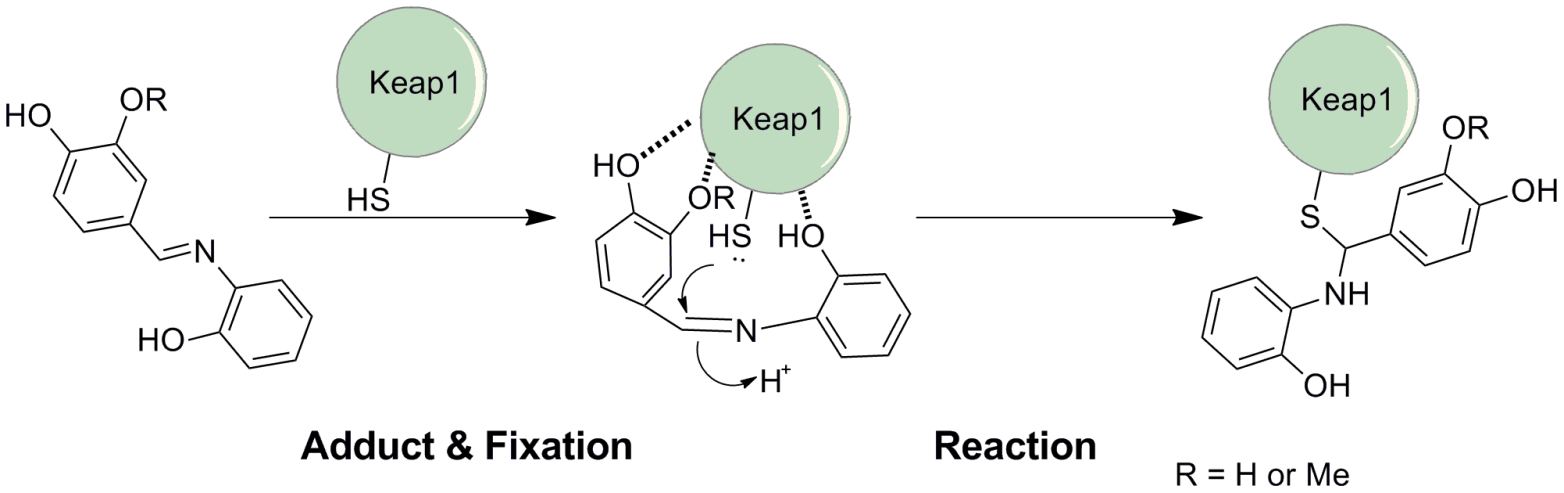
Proposed model for the adduct formation, fixation of IRA, and its reaction with Keap1.

Referring to several examples in which imines react as Michael Acceptors in organic chemistry [32], [33], a plausible mechanism of IRAs adduct Keap1 protein was proposed and shown in **Figure 5**. 6-OH group played a crucial role in incorporating the small molecule to Keap1 on the surface, the 3-OH group and 2-OH/OMe group also performed auxiliary effect to fix IRA molecule to the protein. Thus, the thiol group in cystein residues in the formed complex were in vicinity of the C = N bond. Thiol group then underwent nucleophilic attack to the carbon in imine moiety and a covalent adduct formed as a result. Once Keap1 was modified, Nrf2 could be released.

## Conclusions

In the current study, we have screened the potential of an array of IRAs to be effective Nrf2 activators. Based on SAR analysis, both preliminary and quantitative results were summarized and led us to develop two novel IRAs, 2-methoxyl-3,6-dihydroxyl-IRA and 2,3,6-trihydroxyl-IRA. *In vitro* ARE reporter cell assay indicated that they were both strong Nrf2 activators. A plausible mechanism for their interaction with Keap1 was proposed. Taken together, **33** and **34** are *indirect* antioxidants, which can activate Nrf2-ARE pathway to wake up the defense system in cell. These features warrant further studies in disease models for the development of the IRAs as chemopreventive agents.

## Supporting Information

File S1 **Figure S1.** Compounds **33** and **34** induced ARE-luciferase activities dose-dependently. Table S1. Calculated Descriptors and Corresponding Software Applied. Table S2. Data of Calculated Descriptors. Table S3. Pearson Coefficient Test Results.
(PDF)
