# Supplementary material for: Imine Resveratrol Analogues: Molecular Design, Nrf2 Activation and SAR Analysis
Source: PLoS One. 2014 Jul 16;9(7):e101455. doi: 10.1371/journal.pone.0101455 (PMC4100753; doi:10.1371/journal.pone.0101455)

Nrf2 Inducing Imine Resveratrol Analogs: Molecular Design, *in vitro* Assays and SAR Analysis

Chang Li†, Xiaofei Xu†, Xiu Jun Wang*‡. and Yuanjiang Pan*†

† Department of Chemistry, Zhejiang University, 38 Zheda Road, Hangzhou, 310027, P. R. China

‡ Department of Pharmacology, School of Medicine, Zhejiang University, Hangzhou, 310058, P. R. China

panyuanjiang@zju.edu.cn; xjwang@zju.edu.cn

Supporting information

Table of contents **Page**

I. Synthetic procedure and Spectral Data of IRAs. 2

II. QSAR Analysis. 8

III. Dose-dependence of **33** and **34** 17

I. Synthetic procedure and spectral data of IRAs.

34 imine resveratrol analogues (IRAs, 32 for the 1st-round screening and 2 for later design) were easily prepared as described in the literature with some modification. (see Ref. 15, 18) In brief, the mixture of aromatic aldehyde and aniline were stirred in a small amount of water at room temperature for about 3 hours. Then followed by filtration and recrystallization in EtOAc or MeOH to obtain the pure compounds.

Compound **1**: ^{1}H NMR (500 MHz, $CDCl_3$): 8.47 (s, 1H), 7.92-7.94 (m, 2H), 7.48-7.50 (m, 3H), 7.42 (t, 2H, J = 7.85Hz), 7.23-7.25 (m, 3H); ^{13}C NMR (125 MHz, $CDCl_3$): 160.6, 152.3, 136.4, 131.6, 129.4, 129.0, 129.0, 126.2, 121.1.

Compound **2**: ^{1}H NMR (500 MHz, DMSO-d_6): δ 10.16 (br, 1H), 8.44 (s, 1H), 7.78 (d, 2H, J = 5.0 Hz), 7.38 (s, 2H), 7.20 (s, 3H), 6.99 (d, 2H, J = 10.0 Hz); ^{13}C NMR (125MHz, DMSO-d_6): δ 160.7, 160.0, 152.0, 130.7, 129.2, 127.5, 125.4, 120.9, 115.7.

Compound **3**: ^{1}H NMR (500 MHz, Acetone): δ 8.53 (s, 1H), 7.47 (s, 1H), 7.39 (d, 1H, J = 10.0 Hz), 7.32 (t, 1H, J = 10.0 Hz), 7.27 (d, 2H, J = 10.0 Hz), 3.82 (s, 6H); ^{13}C NMR (125MHz, Acetone): δ 159.4, 158.8, 158.7, 145.7, 139.4, 130.7, 123.2, 121.4, 119.1, 115.2, 55.8.

Compound **4**: ^{1}H NMR (500 MHz, DMSO-d_6): δ 6.98 (d, 2H, J = 7.70Hz), 7.26 (d, 2H, J = 8.15Hz), 7.34-7.39 (m, 2H), 7.41-7.44 (m ,3H, Ar-H), 8.54(s, 1H, CH=N) ,9.77 (s, 1H, OH); ^{13}C NMR (125 MHz, DMSO-d_6): δ 115.2, 115.4, 119.4, 121.6, 121.7, 126.6, 130.0, 130.6, 138.9, 153.0, 158.5, 161.0; HR-ESI-MS: *m/z* $[M+H]^+$ 198.0915 (calcd for $C_{13}H_{11}NO$, 198.0913).

Compound **5**: ^{1}H NMR (500 MHz, $CDCl_3$): δ 6.93 (t, J = 7.55Hz,1H, Ar-H), 7.05 (d, J =7.95Hz,1H, Ar-H), 7.23 (t, J = 7.60Hz,1H, Ar-H), 7.32 (d, J = 7.85Hz,1H, Ar-H), 7.50-7.52 (m,3H, Ar-H), 7.93-7.94 (m, 2H, Ar-H), 8.71 (s,1H, CH=N); ^{13}C NMR (125 MHz, $CDCl_3$): δ 115.2, 116.1, 120.3, 129.0, 129.1, 129.2, 131.9, 135.7, 136.0, 152.5, 157.3; HR-ESI-MS: *m/z* $[M-H]^-$ 196.0766 (calcd for $C_{13}H_{11}NO$, 196.0775).

Compound **6**: ^{1}H NMR (500 MHz, DMSO-d_6): δ 6.88 (t, J = 7.55Hz, 1H, Ar-H),

6.93-6.97 (m, 3H, Ar-H), 7.13 (t, J = 7.55Hz, 1H, Ar-H), 7.35-7.40 (m, 2H, Ar-H), 7.61 (d, J = 7.35Hz ,1H, Ar-H), 8.96 (s, 1H, CH=N), 9.72 (s, 1H, 2'-OH), 13.78 (s, 1H, 2-OH); ^{13}C NMR (125 MHz, DMSO-d_6): δ 116.5, 116.7, 118.7, 119.5, 119.6, 119.6, 128.0, 132.3, 132.8, 134.9, 151.1, 160.7, 161.7; HR-ESI-MS: *m/z* $[M+H]^+$ 214.0864 (calcd for $C_{13}H_{11}NO_2$, 214.0863).

Compound **7**: ^{1}H NMR (500 MHz, $CDCl_3$): δ 6.36 (br, 2H, -OH), 6.93 (t, J = 7.60Hz, 1H, Ar-H), 7.00 (d, J = 6.70Hz, 1H, Ar-H), 7.04 (d, J = 8.00Hz, 1H, Ar-H), 7.22 (d, J = 7.55Hz, 1H, Ar-H), 7.29 (d, J = 7.90Hz, 1H, Ar-H), 7.36 (t, J = 7.80Hz, 1H, Ar-H), 7.41 (d, J = 7.50Hz, 1H, Ar-H), 7.46 (s , H, Ar-H), 8.63 (s, 1H, CH=N); ^{13}C NMR (125 MHz, $CDCl_3$): δ 114.3, 115.3, 116.3, 119.3, 120.5, 122.7, 129.3, 130.4, 135.6, 137.6, 152.3, 156.3, 157.1; HR-ESI-MS: *m/z* $[M+H]^+$ 214.0864 (calcd for $C_{13}H_{11}NO_2$, 214.0863).

Compound **8**: ^{1}H NMR (500 MHz, $CDCl_3$): δ 3.63(s, 3H, $-OCH_3$), 6.77(s, 1H, Ar-H), 6.87(s, 1H, Ar-H), 6.95(d, J = 3.80Hz,1H, Ar-H), 7.07(s, 2H, Ar-H), 7.18(s, 1H, Ar-H), 7.22(s, 1H, Ar-H), 7.30(s, 1H, Ar-H),7.53 (br, 1H, -OH), 8.35(s, 1H, CH=N); ^{13}C NMR (125 MHz, $CDCl_3$): δ 55.1, 112.4, 115.2, 116.3, 117.9, 120.1, 122.1, 128.8, 129.7, 135.6, 137.0, 152.2, 157.4, 159.8 ; HR-ESI-MS: *m/z* $[M+H]^+$ 228.1023 (calcd for $C_{14}H_{13}NO_2$, 228.1019).

Compound **9**: ^{1}H NMR (500 MHz, DMSO-d_6): δ 6.79-6.82 (m, 1H, Ar-H), 6.86-6.89 (m, 3H, Ar-H), 7.01-7.05 (m, 1H, Ar-H), 7.13 (dd, J = 1.15, 7.75Hz, 1H, Ar-H), 7.86 (d, J = 8.50Hz, 2H, Ar-H), 8.54 (s, 1H, CH=N), 8.81 (s, 1H, 2'-OH), 10.12 (s, 1H, 4-OH),; ^{13}C NMR (125 MHz, DMSO-d_6): δ 116.0, 116.2, 119.2, 120.0, 127.1, 128.3, 131.4, 138.8, 151.5, 159.2, 161.0; HR-ESI-MS: *m/z* $[2M+H]^+$ 427.1653 (calcd for $C_{13}H_{11}NO_2$, 427.1653).

Compound **10**: ^{1}H NMR (500 MHz, DMSO-d_6): δ6.58 (s, 1H, Ar-H), 6.61 (d, J = 7.50Hz ,2H, Ar-H), 6.87 (d, J = 8.0 Hz ,2H, Ar-H), 7.15 (t, J = 7.5 Hz, 1H , Ar-H), 7.75 (d, J = 7.5 Hz, 2H , Ar-H), 8.40 (s, 1H, CH=N), 9.44(s, 1H, 3'-OH), 10.09 (s, 1H, 4-OH); ^{13}C NMR (125MHz, DMSO-d_6): δ 108.2, 112.0, 112.9, 116.1, 128.0, 130.2, 131.1, 153.8, 158.5, 160.0, 161.0; HR-ESI-MS: *m/z* $[M+H]^+$ 214.0861 (calcd for $C_{13}H_{11}NO_2$, 214.0863).

Compound **11**: ^{1}H NMR (500 MHz, DMSO-d_6): δ 6.73-6.84 (m, 3H, Ar-H), 6.95-6.98 (m, 2H, Ar-H), 7.24 (t, J = 7.95 Hz ,1H, Ar-H), 7.39-7.42 (m, 1H , Ar-H), 7.64 (dd, J = 7.55,1.35Hz ,1H, Ar-H), 8.89 (s, 1H, CH=N), 9.68 (s, 1H, 3'-OH), 13.15 (s, 1H, 2-OH); ^{13}C NMR (125MHz, DMSO-d_6): δ 108.7, 112.6, 114.6, 117.1, 119.6, 119.8, 130.7, 133.1, 113.8, 149.8, 158.8, 160.8, 163.7; HR-ESI-MS: *m/z* [M+H]$^+$ 214.0868 (calcd for $C_{13}H_{11}NO_2$, 214.0863).

Compound **12**: ^{1}H NMR (500 MHz, DMSO-d_6): δ6.8 (d, J = 8.5Hz, 2H, Ar-H), 7.20 (d, J = 8.45Hz ,2H, Ar-H), 7.49 (d, J = 2.45Hz, 3H, Ar-H), 7.89 (d, J = 2.85Hz, 2H, , Ar-H), 8.61 (s, 1H, CH=N), 9.51 (s, 1H, -OH); ^{13}C NMR (125MHz, DMSO-d_6): δ 116.7, 123.5, 129.2, 129.7, 131.8, 137.4, 143.6, 157.3, 158.2; HR-ESI-MS: *m/z* [M+H]$^+$ 198.0918 (calcd for $C_{13}H_{11}NO$, 198.0913).

Compound **13**: ^{1}H NMR (500 MHz, DMSO-d_6): δ6.84 (d, J = 8.7Hz, 2H, Ar-H), 6.92-6.97(m, 2H, Ar-H), 7.31 (s,1H, Ar-H), 7.32 (s,1H, Ar-H),7.35-7.38 (m, 1H, ,Ar-H), 7.59 (dd, J = 7.65, 1.4Hz,1H, ,Ar-H), 8.90 (s, 1H, CH=N), 9.67(s, 1H, 4'-OH), 13.42 (s, 1H, 2-OH); ^{13}C NMR (125MHz, DMSO-d_6): δ 116.9, 117.4, 119.9, 120.4, 123.6, 133.2, 133.5, 140.2, 157.9, 161.1, 161.2; HR-ESI-MS: *m/z* [M+H]$^+$ 214.0859 (calcd for $C_{13}H_{11}NO_2$, 214.0863).

Compound **14**: ^{1}H NMR (500 MHz, DMSO-d_6): δ6.79 (d, J = 5.6 Hz , 2H, Ar-H), 6.87 (d, J = 5.75Hz ,2H, Ar-H), 7.12 (d, J = 5.75 Hz ,2H, Ar-H), 7.73 (d, J = 5.75 Hz, 2H, , Ar-H), 8.44 (s, 1H, CH=N), 9.43(s, 1H, 4'-OH) ,10.05 (s, 3H, 4-OH); ^{13}C NMR (125MHz, DMSO-d_6): δ 116.6, 116.7, 123.2, 129.0, 131.3, 144.3, 156.7, 157.9, 161.2; HR-ESI-MS: *m/z* [M+H]$^+$ 214.0864 (calcd for $C_{13}H_{11}NO_2$, 214.0863).

Compound **15**: ^{1}H NMR (500 MHz, DMSO-d_6): δ3.80 (s, 6H, -OCH_3), 6.61 (s, 1H, Ar-H), 6.89 (d, J =7.85Hz ,2H, Ar-H), 7.06 (s, 2H, Ar-H), 7.19 (d, J = 7.85 Hz, 2H, , Ar-H), 8.53 (s, 1H, CH=N), 9.53 (s, 1H, -OH); ^{13}C NMR (125MHz, DMSO): δ 56.4, 104.1, 106.9, 116.7, 123.6, 139.5, 143.4, 157.4, 158.1, 161.6; HR-ESI-MS: *m/z* [M+H]$^+$ 258.1124 (calcd for $C_{15}H_{15}NO_3$, 258.1125).

Compound **16**: ^{1}H NMR (500 MHz, DMSO-d_6): δ6.76 (s, 2H, J = 5.0 Hz), 6.81 (d, 1H, J = 5.0 Hz), 7.09 - 7.14 (m, 3H), 7.36 (s, 1H), 8.35 (s, 1H), 9.36 (br, 1H); ^{13}C NMR (125MHz, DMSO-d_6): δ 114.4, 115.9, 116.1, 122.4, 128.9, 143.7, 146.1, 149.1,

156.1, 157.5.

Compound **17**: ^{1}H NMR (500 MHz, DMSO-d_6): δ 6.33 (t, J = 2.25 Hz, 1H, Ar-H), 6.77 (d, J = 2.20Hz ,2H, Ar-H), 7.79 (dd, J =6.60,2.15 Hz ,2H, Ar-H), 7.16 (dd, J =6.60,2.05 Hz ,2H, Ar-H), 8.39 (s, 1H, CH=N), 9.46(s, 2H, 3,5-OH), 9.48 (s, 1H, 4'-OH); ^{13}C NMR (125MHz, DMSO-d_6): δ 105.2, 106.3, 115.6, 122.4, 138.3, 142.5, 156.1, 157.4, 158.6; HR-ESI-MS: *m/z* $[M+H]^+$ 230.0814 (calcd for $C_{13}H_{11}NO_3$, 230.0812).

Compound **18**: ^{1}H NMR (500 MHz, $CDCl_3$): δ 8.25 (s, 1H), 7.24 (d, 2H, J = 10.0 Hz), 7.30 (t, 1H, J = 10.0 Hz), 7.26 (d, 2H, J = 5.0 Hz), 7.18 (t, 1H, J = 10.0 Hz), 6.96 (d, 2H, J = 5.0 Hz), 2.37 (s, 3H); ^{13}C NMR (125MHz, $CDCl_3$): δ 161.3, 156.5, 150.6, 137.2, 130.5, 130.1, 126.9, 126.0, 121.8, 119.3, 118.6, 114.8, 17.9.

Compound **19**: ^{1}H NMR (500 MHz, $CDCl_3$): δ1.47 (s, 3H, $-CH_3$), 2.88 (s, 3H, $-OCH_3$), 5.98 (d, J =7.65Hz , 1H, Ar-H), 6.08 (dd, J =5.75, 2.35Hz, 1H, Ar-H), 6.20 (t, J = 7.35 Hz, 1H, Ar-H), 6.28 (dd, J =7.15, 4.90Hz, 2H, Ar-H), 6.41 (t, J = 7.81 Hz, 1H, Ar-H), 6.49 (d, J = 7.45Hz, 1H, Ar-H), 6.63 (s, 1H, Ar-H), 7.35 (s, 1H, CH=N); ^{13}C NMR (125MHz, $CDCl_3$): δ17.9, 55.3, 112.2, 117.8, 117.8, 122.2, 125.8, 126.8, 129.7, 130.3, 131.9, 137.9, 151.0, 159.3, 160.0; HR-ESI-MS: *m/z* $[M+H]^+$ 226.1228 (calcd for $C_{15}H_{15}NO$, 226.1226).

Compound **20**: ^{1}H NMR (500 MHz, DMSO-d_6): δ 10.18 (br, 1H), 8.43 (s, 1H), 7.77 (d, 2H, J = 10.0 Hz), 7.25 (t, 1H, J = 10.0 Hz), 7.01 (m, 3H), 6.90 (d, 2H, J = 5.0 Hz), 2.32 (s, 3H); ^{13}C NMR (125MHz, DMSO-d_6): δ 161.8, 160.9, 153.2, 139.6, 131.8, 130.2, 127.2, 122.6, 119.2, 116.9, 22.2.

Compound **21**: ^{1}H NMR (500 MHz, DMSO-d_6): δ 10.13 (br, 1H), 8.43 (s, 1H), 7.76 (d, 2H, J = 10.0 Hz), 7.25 (t, 1H, J = 10.0 Hz), 6.97 - 7.01 (m, 3H), 6.88 (d, 2H, J = 10.0 Hz), 2.32 (s, 3H); ^{13}C NMR (125MHz, DMSO-d_6): δ 160.6, 159.8, 152.0, 138.4, 130.6, 128.9, 127.6, 126.0, 121.4, 118.0, 115.6, 21.0.

Compound **22**: ^{1}H NMR (500 MHz, $CDCl_3$): δ 8.44 (s, 1H), 7.54 (s, 1H), 7.37 – 7.43 (m, 2H), 7.30 (t, 1H, J = 10.0 Hz), 7.04 – 7.08 (m, 4H), 3.90 (s, 3H), 2.41 (s, 3H); ^{13}C NMR (125MHz, $CDCl_3$): δ 160.3, 160.2, 152.1, 139.2, 137.9, 129.9, 129.2, 127.0, 122.6, 121.8, 118.5, 118.1, 112.0, 55.6, 21.6.

Compound **23**: ^{1}H NMR (500 MHz, DMSO-d_6): δ 2.32 (s, 3H, -CH_3), 6.88 (d, J =8.45Hz , 2H, Ar-H), 6.97-7.01 (m, 3H, , Ar-H), 7.25 (t, J =7.80Hz ,1H, Ar-H), 7.76 (d, J = 8.50 Hz, 2H, Ar-H), 8.43 (s, 1H, CH=N); ^{13}C NMR (125MHz, DMSO-d_6): δ 22.0, 116.6, 119.0, 122.4, 127.0, 128.5, 129.9, 131.6, 139.4, 153.0, 160.7, 161.6; HR-ESI-MS: *m/z* $[M+H]^+$ 212.1071 (calcd for $C_{14}H_{13}NO$, 212.1070).

Compound **24**: ^{1}H NMR (500 MHz, DMSO-d_6): δ 10.12 (s, 1H), 8.44 (s, 1H), 7.76 (d, 2H, J = 10.0 Hz), 7.18 (d, 2H, J = 10.0 Hz), 7.11 (d, 2H, J = 10.0 Hz), 6.88 (d, 2H, J = 10.0 Hz), 2.30 (s, 3H); ^{13}C NMR (125MHz, DMSO-d_6): δ 160.5, 159.1, 149.4, 134.6, 127.6, 120.8, 115.6, 20.6.

Compound **25**: ^{1}H NMR (500 MHz, DMSO-d_6): δ 2.31 (s, 3H, -CH_3), 6.92 (d, J =7.16Hz , 2H, Ar-H), 7.16 (d, J =8.10Hz ,2H, Ar-H), 7.20 (d, J = 8.00 Hz, 2H, Ar-H), 7.29-7.33 (m, 2H, , Ar-H), 7.36 (s, 1H, Ar-H), 8.51 (s, 1H, CH=N), 9.70 (s, 1H, -OH); ^{13}C NMR (125MHz, DMSO-d_6): δ 21.6, 115.2, 119.6, 121.2, 122.0, 130.7, 130.8, 136.3, 138.5, 149.8, 158.7, 160.7; HR-ESI-MS: *m/z* $[M+H]^+$ 212.1071 (calcd for $C_{14}H_{13}NO$, 212.1070).

Compound **26**: ^{1}H NMR (500 MHz, $CDCl_3$): δ 2.17 (s, 3H, -CH_3), 3.64 (s, 3H, -OCH_3), 6.86 (dd, J =7.90,1.70Hz , 1H, Ar-H), 6.97-7.01 (m, 4H, Ar-H), 7.15 (t, J = 7.65Hz, 1H, Ar-H), 7.20 (d, J =7.40Hz ,1H, Ar-H), 7.35 (s, 1H, Ar-H), 8.21 (s, 1H, CH=N); ^{13}C NMR (125MHz, $CDCl_3$): δ 21.0, 55.3, 111.8, 118.1, 120.9, 122.3, 129.7, 129.8, 135.8, 137.8, 149.3, 159.5, 160.0; HR-ESI-MS: *m/z* $[M+H]^+$ 226.1232 (calcd for $C_{15}H_{15}NO$, 226.1226).

Compound **27**: ^{1}H NMR (500 MHz, $CDCl_3$): δ 8.43 (s, 1H), 7.84 (d, 2H, J = 10.0 Hz), 7.44 (d, 2H, J = 5.0 Hz), 7.22 (d, 2H, J = 10.0 Hz), 7.16 (d, 2H, J = 10.0 Hz), 2.40 (s, 3H); ^{13}C NMR (125MHz, $CDCl_3$): δ 158.0, 149.1, 137.2, 136.2, 134.9, 129.9, 129.1, 120.9, 21.1.

Compound **28**: ^{1}H NMR (500 MHz, $CDCl_3$): δ 3.84 (s, 3H, 4-OCH_3), 3.88 (s, 3H, 4'-OCH_3), 6.93 (d, J =8.85Hz , 2H, Ar-H), 6.99(d, J =8.70Hz ,2H, Ar-H), 7.22 (d, J = 8.80 Hz, 2H, Ar-H), 7.84(d, J =8.70Hz ,2H, Ar-H), 8.41 (s, 1H, CH=N); ^{13}C NMR (125MHz, $CDCl_3$): δ 55.6, 55.7, 114.4, 114.6, 122.3, 129.7, 130.5, 145.5, 158.1, 158.1, 162.2; HR-ESI-MS: *m/z* $[M+H]^+$ 242.1175 (calcd for $C_{15}H_{15}NO_2$, 242.1176).

Compound **29**: ^{1}H NMR (500 MHz, $CDCl_3$): δ 3.83 (s, 3H), 3.91 (s, 3H), 3.94 (s, 6H), 6.93 (d, 2H, J = 10.0 Hz), 7.14 (s, 2H), 7.22 (d, 2H, J = 10.0 Hz), 8.37 (s, 1H); ^{13}C NMR (125MHz, $CDCl_3$): δ 55.6, 56.4, 61.1, 105.7, 114.5, 122.3, 132.1, 140.8, 145.0, 153.6, 158.1, 158.4.

Compound **30**: ^{1}H NMR (500 MHz, $CDCl_3$): δ 3.91 – 3.95 (m, 12H), 5.67 (s, 1H), 6.78 (d, 1H, J = 0), 6.80 (m, 2H), 7.14 (s, 2H), 8.35 (s, 1H); ^{13}C NMR (125MHz, $CDCl_3$): δ 56.3, 56.4, 61.1, 105.8, 107.2, 111.0, 113.5, 132.0, 140.9, 145.4, 153.7, 158.5.

Compound **31**: ^{1}H NMR (500 MHz, DMSO-d_6): δ 3.65 (s, 3H), 3.81 (s, 6H), 3.84 (s, 3H), 6.56 (s, 2H), 7.04 (d, 1H, J = 10.0 Hz), 7.30 (d, 1H, J = 5.0 Hz), 7.40 (d, 1H, J = 0), 8.47 (s, 1H), 9.31 (s, 1H); ^{13}C NMR (125MHz, DMSO-d_6): δ 55.6, 55.9, 60.1, 98.5, 111.6, 113.6, 122.2, 129.2, 135.5, 146.7, 147.5, 150.8, 153.2, 159.4.

Compound **32**: ^{1}H NMR (500 MHz, $CDCl_3$): δ 8.29 (s, 1H), 7.64 (d, 1H, J = 10.0 Hz), 7.52 (s, 1H), 7.42 (d, 1H, J = 10.0 Hz), 7.32 – 7.36 (m, 2H), 7.09 (t, 1H, J = 10.0 Hz), 7.03 (t, 2H, J = 10.0 Hz); ^{13}C NMR (125MHz, $CDCl_3$): δ 162.3, 156.3, 150.4, 133.2, 130.3, 128.6, 127.0, 119.6, 114.7.

Compound **33**: ^{1}H NMR (500 MHz, DMSO-d_6): δ 3.91 (s, 3H), 6.85 (t, 1H, J = 10.0 Hz), 6.95 (d, 2H, J = 10.0 Hz), 7.10 (t, 1H, J = 0), 7.20 (d, 1H, J = 0), 7.80 (s, 1H), 8.59 (s, 1H), 8.91 (s, 1H), 9.76 (s, 1H); ^{13}C NMR (125MHz, DMSO-d_6): δ 55.8, 110.8, 115.3, 118.6, 119.6, 124.7, 127.0, 128.4, 138.1, 148.2, 150.3, 151.3, 158.8.

Compound **34**: ^{1}H NMR (500 MHz, DMSO-d_6): δ 6.80 (t, 1H, J = 10.0 Hz), 6.86 (t, 2H, J = 10.0 Hz), 7.01 (t, 1H, J = 5.0 Hz), 7.10 (d, 1H, J = 5.0 Hz), 7.25 (d, 1H, J = 5.0 Hz), 7.47 (s, 1H), 8.44 (s, 1H), 8.82 (s, 1H), 9.24 (br, 1H), 9.58 (br, 1H); ^{13}C NMR (125MHz, DMSO-d_6): δ 114.8, 115.4, 115.7, 119.0, 119.5, 122.5, 126.5, 128.4, 138.7, 145.5, 149.1, 150.8, 159.2.

II. Theoretical Calculation of QSAR Descriptors.

Table S1. Calculated Descriptors and Corresponding Software Applied

Descriptor	Software Applied
Vertical Detachment Energy (**IP**/kJ•mol^{-1}), Vertical Electronic Affinity Energy (**EA**/kJ•mol^{-1}), Chemical Potential (**μ**/kJ•mol^{-1}), Dipole Moment (**DM**/Debye), Chemical Hardness (**η**/kJ•mol^{-1}), Chemical Softness (**S**/kJ•mol^{-1}), Electrophilicity (**ω**/kJ•mol^{-1}), Energy of the Lowest Unoccupied Molecular Orbital ($\mathbf{E_{LUMO}}$/Hartree), Energy of the Highest Occupied Molecular Orbital ($\mathbf{E_{HOMO}}$/Hartree), Energy Gap between LUMO and HOMO ($\mathbf{\Delta E_{L\text{-}H}}$/Hartree), Bond Length of C=N ($\mathbf{L_{C=N}}$/A), APT Charge of C in C=N (**qCA**/e), APT Charge of N in C=N (**qNA**/e), Electrophilic Frontier Electronic Density ($\mathbf{Fr^{E}}$), Nucleophilic Frontier Electronic Density ($\mathbf{Fr^{N}}$), Radical Frontier Electronic Density ($\mathbf{Fr^{R}}$)	Gaussian 09W<br>(the structure was first optimized using DFT at the B3LYP/6–31 + G(d) level, the descriptors were then obtained directly or generated using frequency calculation or pop analysis)
Molecular Weight (**MW**), **logP**, Calculated logP (**clogP**), Molar Refractivity (**MR**/mL•mol^{-1}), Calculated Molar Refractivity (**cMR**/mL•mol^{-1}), Topological Polar Surface Area (**tPSA**), Heat of Formation ($\mathbf{H_{fs}}$/kJ•mol^{-1}), Henry's Law Constant (**pHLC**)	ChemBioDraw Ultra 12.0

Table S2. Data of Calculated Descriptors (Part 1, **1-5**)

Compound	**1**	**2**	**3**	**4**	**5**
MW	181	197	241	197	197
IP/kJ•mol^{-1}	739.7183	728.9233	708.248	738.4582	714.6225
EA/kJ•mol^{-1}	-16.7916	4.252969	28.20094	-20.7369	-6.64716
**μ**/kJ•mol^{-1}	-361.463	-366.588	-368.224	-358.861	-353.988
**η**/kJ•mol^{-1}	378.2549	362.3352	340.0235	379.5975	360.6348
**S**/kJ•mol^{-1}	0.001322	0.00138	0.00147	0.001317	0.001386
**ω**/kJ•mol^{-1}	172.7086	185.4455	199.3822	169.6283	173.7315
**DM**/Debye	2.7544	3.6865	1.1312	1.7559	2.5957
$\mathbf{E_{HOMO}}$/Hartree	-0.22025	-0.21744	-0.20966	-0.22121	-0.21069
$\mathbf{E_{LUMO}}$/Hartree	-0.06591	-0.05767	-0.05056	-0.06702	-0.0622
$\mathbf{\Delta E_{L-H}}$/Hartree	0.15434	0.15977	0.1591	0.15419	0.14849
$\mathbf{L_{C=N}}$/A	1.27828	1.27932	1.27692	1.27776	1.27699
**qCA**/e	0.647	0.756	0.68	0.642	0.662
**qNA**/e	-0.757	-0.843	-0.778	-0.751	-0.747
**logP**	3.9	3.51	3.65	3.51	3.51
**clogP**	2.85	3.084	3.3	3.084	2.183
**tPSA**	12.36	32.59	30.82	32.59	32.59
**MR**/mL•mol^{-1}	58.8	60.62	73.3	60.62	60.62
**cMR**/mL•mol^{-1}	5.8549	6.008	7.0887	6.008	6.008
$\mathbf{H_{fs}}$/kJ•mol^{-1}	243.63	66.32	-85.03	66.32	66.32
**pHLC**	1.25	2	2.25	2	1.25
$\mathbf{Fr^E}$	2.799471	2.506731	0.730933	2.618258	6.574901
$\mathbf{Fr^N}$	8.057701	10.18686	1.001542	9.77145	11.08404
$\mathbf{Fr^R}$	10.85717	12.69359	1.732475	12.38971	17.65894

(Part 2, **6-10**)

Compound	**6**	**7**	**8**	**9**	**10**
MW	213	213	227	213	243
IP/kJ•mol^{-1}	729.0065	715.4668	707.1811	702.9894	683.0151
EA/kJ•mol^{-1}	-21.0988	-10.6358	-9.67077	7.256016	-10.7709
**μ**/kJ•mol^{-1}	-353.954	-352.416	-348.755	-355.123	-336.122
**η**/kJ•mol^{-1}	375.0526	363.0513	358.4259	347.8667	346.893
**S**/kJ•mol^{-1}	0.001333	0.001377	0.001395	0.001437	0.001441
**ω**/kJ•mol^{-1}	167.021	171.0457	169.6727	181.265	162.8428
**DM**/Debye	2.0645	1.2391	2.3894	3.3625	3.5547
$\mathbf{E_{HOMO}}$/Hartree	-0.21695	-0.21168	-0.20988	-0.20789	-0.20715
$\mathbf{E_{LUMO}}$/Hartree	-0.06543	-0.06334	-0.0609	-0.05523	-0.05826
$\mathbf{\Delta E_{L-H}}$/Hartree	0.15152	0.14834	0.14898	0.15266	0.14889
$\mathbf{L_{C=N}}$/A	1.27925	1.27644	1.27817	1.27942	1.28174
**qCA**/e	0.58	0.657	0.657	0.76	0.731
**qNA**/e	-0.631	-0.742	-0.746	-0.816	-0.717
**logP**	3.12	3.12	3.39	3.12	3
**clogP**	2.417	2.417	2.527	2.417	2.27657
**tPSA**	52.82	52.82	41.82	52.82	62.05
**MR**/mL•mol^{-1}	62.43	62.43	67.87	62.43	69.68
**cMR**/mL•mol^{-1}	6.1611	6.1611	6.6249	6.1611	6.778
$\mathbf{H_{fs}}$/kJ•mol^{-1}	-110.99	-110.99	-98.01	-110.99	-275.32
**pHLC**	2	2	1.75	2	2.5
$\mathbf{Fr^E}$	9.328548	6.561068	5.451692	5.023544	7.699287
$\mathbf{Fr^N}$	26.22864	13.33409	11.73239	10.69154	35.74431
$\mathbf{Fr^R}$	35.55719	19.89515	17.18408	15.71508	43.4436

(Part 3, **11-15**)

Compound	**11**	**12**	**13**	**14**	**15**
MW	229	213	213	197	213
IP/kJ•mol^{-1}	692.6097	719.1865	741.166	705.3008	720.1306
EA/kJ•mol^{-1}	-15.6466	-21.4066	-51.2397	-16.194	-29.8641
**μ**/kJ•mol^{-1}	-338.482	-348.89	-344.963	-344.553	-345.133
**η**/kJ•mol^{-1}	354.1281	370.2965	396.2028	360.7474	374.9973
**S**/kJ•mol^{-1}	0.001412	0.00135	0.001262	0.001386	0.001333
**ω**/kJ•mol^{-1}	161.7632	164.3604	150.1751	164.5432	158.8237
**DM**/Debye	3.1856	3.4736	2.0093	3.4083	4.5759
$\mathbf{E_{HOMO}}$/Hartree	-0.20985	-0.21951	-0.22744	-0.20717	-0.20732
$\mathbf{E_{LUMO}}$/Hartree	-0.06082	-0.06329	-0.07485	-0.06484	-0.06222
$\mathbf{\Delta E_{L\text{-}H}}$/Hartree	0.14903	0.15622	0.15259	0.14233	0.1451
$\mathbf{L_{C=N}}$/A	1.28143	1.28224	1.28239	1.27965	1.28193
**qCA**/e	0.711	0.724	0.666	0.574	0.42
**qNA**/e	-0.69	-0.739	-0.677	-0.647	-0.499
**logP**	2.73	3.12	3.12	3.51	3.12
**clogP**	2.0903	2.417	2.417	2.183	2.417
**tPSA**	73.05	52.82	52.82	32.59	52.82
**MR**/mL•mol^{-1}	64.25	62.43	62.43	60.62	62.43
**cMR**/mL•mol^{-1}	6.3142	6.1611	6.1611	6.008	6.1611
$\mathbf{H_{fs}}$/kJ•mol^{-1}	-288.3	-110.99	-110.99	66.32	-110.99
**pHLC**	2.75	2	2	1.25	2
$\mathbf{Fr^E}$	12.21033	1.465971	1.274518	1.696288	1.881708
$\mathbf{Fr^N}$	39.08066	27.13677	36.32557	10.35663	27.55947
$\mathbf{Fr^R}$	51.29099	28.60274	37.60009	12.05292	29.44118

(Part 4, **16-20**)

Compound	**16**	**17**	**18**	**19**	**20**
MW	213	257	197	229	211
IP/kJ•mol^{-1}	691.8489	698.5413	686.231	729.1795	725.0757
EA/kJ•mol^{-1}	-15.7462	-17.1286	-19.0884	-340.193	-21.4603
**μ**/kJ•mol^{-1}	-338.051	-340.706	-333.571	-194.493	-351.808
**η**/kJ•mol^{-1}	353.7975	357.835	352.6597	534.6863	373.268
**S**/kJ•mol^{-1}	0.001413	0.001397	0.001418	0.000935	0.00134
**ω**/kJ•mol^{-1}	161.503	162.1988	157.7581	35.37367	165.7906
**DM**/Debye	4.0154	1.9984	3.2427	4.1347	1.731
$\mathbf{E_{HOMO}}$/Hartree	-0.20805	-0.20767	-0.23053	-0.22214	-0.21723
$\mathbf{E_{LUMO}}$/Hartree	-0.06107	-0.06287	-0.07682	-0.07636	-0.06663
$\mathbf{\Delta E_{L-H}}$/Hartree	0.14698	0.1448	0.15371	0.14578	0.1506
$\mathbf{L_{C=N}}$/A	1.28283	1.27968	1.28254	1.29141	1.27781
**qCA**/e	0.618		0.627	0.458	0.625
**qNA**/e	-0.147		-0.565	-0.422	-0.71
**logP**	3.12	3.26	2.73	2.73	4
**clogP**	2.417	2.633	2.0903	2.0203	3.583
**tPSA**	52.82	51.05	73.05	73.05	32.59
**MR**/mL•mol^{-1}	62.43	75.12	64.25	64.25	66.52
**cMR**/mL•mol^{-1}	6.1611	7.2418	6.3142	6.3142	6.4718
$\mathbf{H_{fs}}$/kJ•mol^{-1}	-110.99	-262.34	-288.3	-288.3	34.21
**pHLC**	2	2.25	2.75	2.75	2
$\mathbf{Fr^{E}}$	2.907351	3.281355	2.882836	2.758912	3.535548
$\mathbf{Fr^{N}}$	29.1893	14.04516	37.23451	26.95615	17.8173
$\mathbf{Fr^{R}}$	32.09665	17.32651	40.11735	29.71506	21.35285

(Part 5, **21-25**)

Compound	**21**	**22**	**23**	**24**	**25**
MW	225	211	211	225	211
IP/kJ•mol^{-1}	717.0493	713.87	731.8239	717.7549	716.7023
EA/kJ•mol^{-1}	-16.7825	1.917088	-39.483	-14.4549	5.384717
**μ**/kJ•mol^{-1}	-350.133	-357.894	-346.17	-351.65	-361.044
**η**/kJ•mol^{-1}	366.9159	355.9765	385.6534	366.1049	355.6588
**S**/kJ•mol^{-1}	0.001363	0.001405	0.001297	0.001366	0.001406
**ω**/kJ•mol^{-1}	167.0592	179.9105	155.3648	168.8829	183.2549
**DM**/Debye	2.5424	3.6438	1.8681	2.2185	3.4347
$\mathbf{E_{HOMO}}$/Hartree	-0.21511	-0.21339	-0.2244	-0.21572	-0.21426
$\mathbf{E_{LUMO}}$/Hartree	-0.06403	-0.05765	-0.0701	-0.06302	-0.05645
$\mathbf{\Delta E_{L\text{-}H}}$/Hartree	0.15108	0.15574	0.1543	0.1527	0.15781
$\mathbf{L_{C=N}}$/A	1.278	1.2795	1.28064	1.27797	1.27923
**qCA**/e	0.642	0.733	0.562	0.648	0.745
**qNA**/e	-0.738	-0.795	-0.601	-0.766	-0.834
**logP**	4.26	4	4	4.26	4
**clogP**	3.693	3.583	3.583	3.693	3.583
**tPSA**	21.59	32.59	32.59	21.59	32.59
**MR**/mL•mol^{-1}	71.95	66.52	66.52	71.95	66.52
**cMR**/mL•mol^{-1}	6.9356	6.4718	6.4718	6.9356	6.4718
$\mathbf{H_{fs}}$/kJ•mol^{-1}	47.19	34.21	34.21	47.19	34.21
**pHLC**	1.75	2	2	1.75	2
$\mathbf{Fr^E}$	3.386861	3.551588	2.844179	5.265173	4.232319
$\mathbf{Fr^N}$	19.28362	24.69562	41.5246	17.45218	18.47296
$\mathbf{Fr^R}$	22.67048	28.24721	44.36878	22.71736	22.70528

(Part 6, **26-30**)

Compound	**26**	**27**	**28**	**29**	**30**
MW	211	211	225	230	241
IP/kJ•mol^{-1}	706.9135	717.9727	709.4381	727.644	678.8547
EA/kJ•mol^{-1}	4.685887	-18.4157	-14.7425	-39.9474	6.171527
**μ**/kJ•mol^{-1}	-355.8	-349.779	-347.348	-343.848	-342.513
**η**/kJ•mol^{-1}	351.1138	368.1942	362.0903	383.7957	336.3416
**S**/kJ•mol^{-1}	0.001424	0.001358	0.001381	0.001303	0.001487
**ω**/kJ•mol^{-1}	180.2741	166.142	166.6028	154.0294	174.3989
**DM**/Debye	3.3481	1.4434	2.2193	0.8071	4.5328
$\mathbf{E_{HOMO}}$/Hartree	-0.21085	-0.21462	-0.21261	-0.2185	-0.20095
$\mathbf{E_{LUMO}}$/Hartree	-0.05641	-0.06538	-0.06275	-0.0723	-0.05456
$\mathbf{\Delta E_{L\text{-}H}}$/Hartree	0.15444	0.14924	0.14986	0.1462	0.14639
$\mathbf{L_{C=N}}$/A	1.27965	1.27798	1.27851	1.27837	1.27564
**qCA**/e	0.734	0.627	0.625	0.648	0.731
**qNA**/e	-0.824	-0.739	-0.741	-0.74	-0.795
**logP**	4	4	4.26	4.95	3.65
**clogP**	3.583	3.583	3.693	4.062	3.2167
**tPSA**	32.59	32.59	21.59	12.36	30.82
**MR**/mL•mol^{-1}	66.52	66.52	71.95	69.31	73.3
**cMR**/mL•mol^{-1}	6.4718	6.4718	6.9356	6.8101	7.0887
$\mathbf{H_{fs}}$/kJ•mol^{-1}	34.21	34.21	47.17	184.32	-85.03
**pHLC**	2	2	1.75	1.38	1.75
$\mathbf{Fr^E}$	3.374047	3.065443	5.467477	4.90188	1.286592
$\mathbf{Fr^N}$	17.66091	15.87043	15.80724	12.24535	16.79908
$\mathbf{Fr^R}$	21.03496	18.93587	21.27472	17.14723	18.08567

(Part 7, **31-34**)

Compound	**31**	**32**	**33**	**34**
MW	301	317	317	275
IP/kJ•mol^{-1}	684.1958	667.7081	690.1196	744.8342
EA/kJ•mol^{-1}	-13.6225	-24.6781	-15.7978	-58.2299
**μ**/kJ•mol^{-1}	-335.287	-321.515	-337.161	-343.302
**η**/kJ•mol^{-1}	348.9091	346.1931	352.9587	401.532
**S**/kJ•mol^{-1}	0.001433	0.001444	0.001417	0.001245
**ω**/kJ•mol^{-1}	161.098	149.298	161.0351	146.7584
**DM**/Debye	3.3543	3.7493	4.4358	3.2427
$\mathbf{E_{HOMO}}$/Hartree	-0.2042	-0.20442	-0.20735	-0.23053
$\mathbf{E_{LUMO}}$/Hartree	-0.05963	-0.0613	-0.06197	-0.07682
$\mathbf{\Delta E_{L-H}}$/Hartree	0.14457	0.14312	0.14538	0.15371
$\mathbf{L_{C=N}}$/A	1.28001	1.28208	1.27854	1.2799
**qCA**/e		0.633	0.73	0.655
**qNA**/e		-0.61	-0.837	-0.668
**logP**	3.4	3.01	3.01	4.34
**clogP**	2.60553	1.79614	2.2262	4.1178
**tPSA**	49.28	69.51	69.51	32.59
**MR**/mL•mol^{-1}	87.8	89.62	89.62	68.31
**cMR**/mL•mol^{-1}	8.3225	8.4756	8.4756	6.785
$\mathbf{H_{fs}}$/kJ•mol^{-1}	-413.69	-591	-591	81.18
**pHLC**	2.76	2.76	2.5	2
$\mathbf{Fr^E}$	3.192434	2.441696	3.909881	4.31848
$\mathbf{Fr^N}$	38.40461	58.98494	24.30078	55.13277
$\mathbf{Fr^R}$	41.59704	61.42664	28.21066	59.45125

Table S3. Pearson Coefficient Test Results

Descriptor	Coefficient	Descriptor	Coefficient	Descriptor	Coefficient
**MW**	.088	**E_{HOMO}**	.252	**tPSA**	-.159
**IP**	-.217	**E_{LUMO}**	.255	**MR**	.187
**EA**	.092	**ΔE**	-.044	**CMR**	.179
**μ**	-.017	**$L_{C=N}$**	-.200	**H_{fs}**	.011
**η**	-.147	**qCA**	.136	**pHLC**	-.073
**S**	.188	**qNA**	-.196	**FrE**	-.035
**ω**	.093	**logP**	.162	**FrN**	-.106
**DM**	.220	**clogP**	.167	**FrR**	-.108

III. Dose-dependence of **33** and **34**

Figure S1. Compounds **33** and **34** induced ARE-luciferase activities dose-dependently. ARE reporter cells were exposed to the compound **33** or the compound **34 (**1.88 to 30 μM) for 24 h before the luciferase activities were measured. The value for cells treated with vehicle DMSO (0.1% v/v) was set at 1. Results are from three separate experiments.

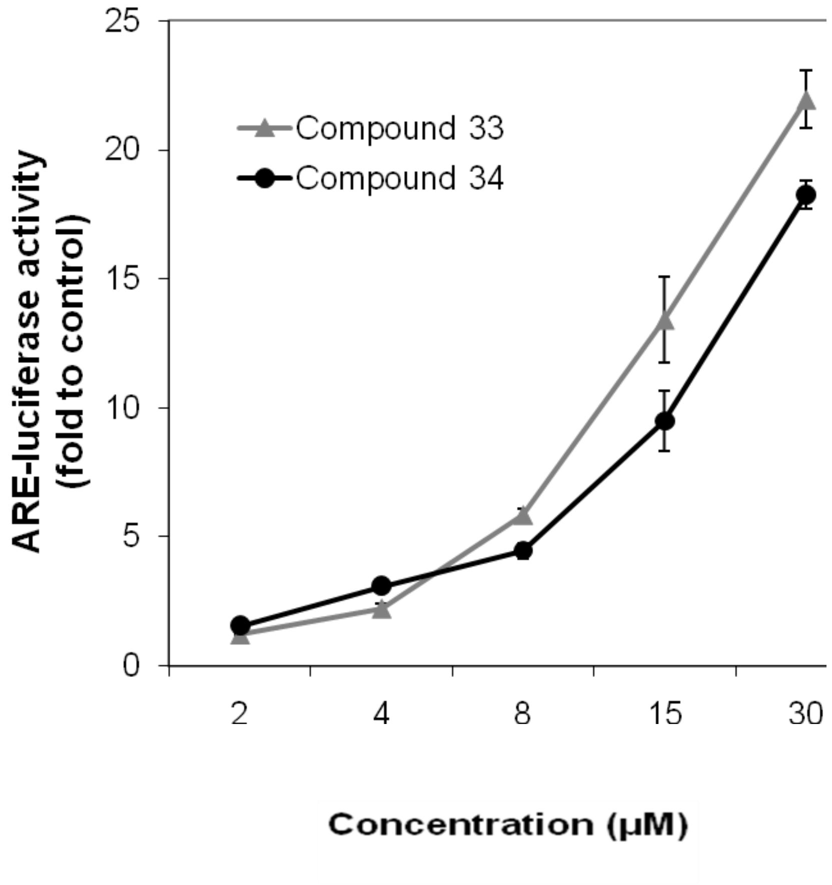

Supplement: File S1 — Figure S1. Compounds 33 and 34 induced ARE-luciferase activities dose-dependently. Table S1. Calculated Descriptors and Corresponding Software Applied. Table S2. Data of Calculated Descriptors. Table S3. Pearson Coefficient Test Results. (PDF) [file pone.0101455.s001.pdf]
